# Highly biocompatible material for enhanced abdominal wall repair: a retrospective study with EGIS^®^ porcine dermal matrix

**DOI:** 10.1080/23320885.2023.2285054

**Published:** 2023-11-22

**Authors:** Franco Bassetto, Tito Brambullo, Bernardo Biffoli, Nicola Baldan, Marco Rastrelli, Simone Mocellin, Vincenzo Vindigni

**Affiliations:** a Department of Neuroscience: Neurological, Psychiatric, Sensorial, Reconstructive, and Rehabilitative Sciences, University of Padua, Padua, Italy; b Department of Surgical, Oncological and Gastroenterological Sciences, University of Padua, Padua, Italy; c Surgical Oncology Unit, Veneto Institute of Oncology, IOV-IRCCS, Padua, Italy

**Keywords:** Acellular dermal matrix, trunk surgery, soft tissue reinforcement

## Abstract

In the early 2000s, medical devices based on acellular matrices multiplied in number. Nowadays, the use of porcine ADMs is to be considered a well-established technology, commonly applied in different surgical specialties. In this retrospective analysis of 110 cases, the use of non-crosslinked porcine ADM EGIS^®^ results a safe and effective tool in many procedures and specialties.

## Introduction

Surgical reconstruction of the abdominal wall, as in cases of hernias and other defects, is performed with different modalities depending on each patient’s clinical needs, as well as hospital centres possibilities, and preferences of operating surgeons.

In the case of a hernia, the abdominal wall has lost its integrity or part of its ability to hold intra-abdominal pressures. To resolve this pathological weakness, different techniques are applied, and multiple medical devices are available to support patients’ tissues.

The application of reconstructive materials for soft tissues defects is reported since 1910s with the use of autologous or xenogeneic tissues like *fascia lata*, and later also as preserved derivatives [1,2].

Before the development of plastic materials and their standardized production processes, surgical supports in the repair of abdominal, inguinal, and thoracic walls defects comprised biological tissues of different kinds, textile fibres like cotton, or metal nets made of tantalum or stainless-steel alloys [3,4]. After the second World War, plastic polymers industry started proposing a plethora of nets and meshes eliciting different reactions when implanted [5,6].

In the 1980s the application of processed biological products entered common clinical practice and expands to other specialties and techniques [7]. With the industrialization of the production process of cadaveric or xenogeneic freeze-dried tissues, actual medical devices with standardized characteristics have been developed [8,9].

For the use in general surgery in combination with synthetic meshes in abdominal wall repair (AWR), it was demonstrated that the biological dermal matrix prevents synthetic polypropylene mesh from adhering to viscera. It was also demonstrated the effective clinical applicability of ADM alone as a support for abdominal wall reconstruction surgery [10,11].

In the early 2000s, medical devices based on acellular dermal matrices multiplied in number, either from cadaveric or xenogeneic dermis, presenting very different biochemical properties. Indeed, it is possible to distinguish devices with different degrees of collagen fibres integrity, and devices in which collagen has been processed with chemical reagents in order to produce crosslinks, as well as devices containing preservatives.

Nowadays, the use of porcine acellular dermal matrices appears to be considered a well-established technology, with a consolidated role in trunk surgery, among other disciplines, for the reconstruction, reinforcement, repair, and substitution of soft tissues in plastic and reconstructive surgery applications. We decided to adopt the use of the porcine acellular dermal matrix EGIS^®^ to confirm this hypothesis, and here we report our experience using this ADM.

## Materials and methods

Cases of implantation of biological matrix EGIS^®^ from two Italian centres were retrospectively collected. Surgeries were performed following general practice guidelines for surgical membranes implantation and the collection encompassed all procedures of plastic and reconstructive surgery and general surgery performed, with no exclusion on reasons for surgery. The study was conducted in accordance with the Declaration of Helsinki, prepared in accordance with the STROBE checklist, and written consent has been obtained from patients. Ethical approval was not required given the retrospective nature of the analysis.

### Data collection

The retrospective data collection was performed on a structured spreadsheet (Microsoft Excel, Microsoft Corporation, One Microsoft Way, Redmond, WA). Two hospital centres established in the same town took part to the data collection: one University Hospital with a multidisciplinary approach to surgical treatment, and a specialized Regional Oncological Centre focussed on the treatment of neoplastic conditions. All surgeries performed using the porcine acellular dermal matrix EGIS^®^ (Audio Technologies, Piacenza, IT; licensed by DECOMED, Venice, IT) as soft tissue substitute were recorded in order to assess its clinical outcomes, including effectiveness and complication rates. Records were checked for consistency, excluding from the analysis those records lacking fundamental data and those in which the available follow-up was shorter than 3 months. A total of 110 entries were considered for the analysis.

The EGIS^®^-assisted surgical procedures collected on the spreadsheet were performed between January 2021 and March 2023. Demographic data for all 110 patients were collected and sex, age, BMI, Ventral Hernia Working Group (VHWG) grades, and reason for surgery were specified. Available surgical details were recorded, along with possible previous surgeries and implants as well. In addition, surgeries were distinguished for priority (elective or emergency) and type (open or laparoscopic). Post-operative complications such as seroma, infection, hematoma, necrosis, and dehiscence were checked. Attention was posed to defect recurrence and the need for reintervention. Data were analysed and presented using basic descriptive statistic tools.

### EGIS^®^ acellular dermal matrix

Acellular dermal matrices (ADMs) have been in use in clinical practice for almost three decades now. EGIS^®^ is an ADM of porcine origin, available in different sizes with a thickness of either 0.8 or 1.5 mm. It is completely natural, without preservatives and crosslinking agents, and it allows cells colonisation and vascular invasion leading to the regeneration of the matrix into patient’s new tissue.

### Surgery

All surgeries were performed using EGIS^®^ ADM in the role of tissue substitute. EGIS^®^ matrix is supplied dry and must be hydrated for about 10–15 min in sterile saline solution before use. Various sizes of EGIS^®^ were used, depending on the type, size, and position of the specific patient’s tissue defect. Surgeries were carried out following these main steps, with variations on the details depending on the specific case.

**Figure 1.**
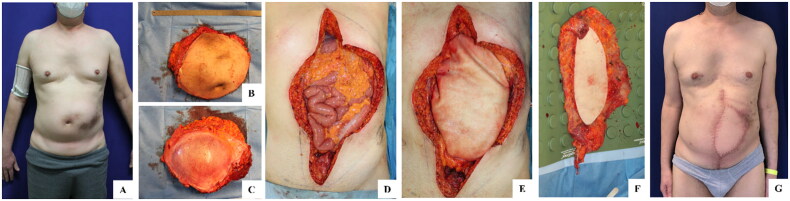
A case of functional abdominal reconstruction after retroperitoneal sarcoma resection. (A) before surgery; (B,C) the macroscopical aspect of the sarcoma; (D) the abdominal wall defect; (E) reconstruction of the abdominal wall with EGIS^®^ porcine dermal matrix, and (F) innervated latissimus dorsii muscolocutaneous free flap; (G) the final results.

1. Surgical site preparation with removal of infected substance or necrotic tissue, and removal of possible previous implants;
2. Determination of the size and extension of the defect and of available autologous tissue for defect coverage to determine the operative plan and the size of membrane needed;
3. Membrane hydration in saline solution, and trimming for implantation site adaptation when suitable;
4. Matrix suturing and drains insertion (an example of intra-operative images can be seen in Figure 1(B–F)).

EGIS^®^ was sutured to the surrounding tissues where an overlap of at least 1 cm was created to ensure stability of the matrix and its intimate contact with living tissues.

## Results

Cases from our centres were retrospectively reviewed, including a time span going from January 2021 to March 2023. In this period, the overall number of procedures with EGIS^®^ ADM (Audio Technologies) were 113, 3 of them were followed by other centres, thus 110 patients were included in the analysis.

Data were retrieved for the operations regarding trunk surgery, in particular for those operated by the plastic and reconstructive surgery unit and the oncological surgery unit. It was possible to gather complete and up-to-date information regarding 110 patients, with a follow-up of 13.2(±8.1) months.

A summarised in Table 1, the examined group had an average age of 59.8 (±16.5) years and presented a trunk defect mean size of 135 cm^2^. Specific implantation of EGIS was necessary for: abdominal wall repair (82 cases), parastomal hernia repair (6 cases), inguinal hernia repair (18 cases), repair of other trunk and limbs defects (9 cases). Five patients received multiple implantations since they presented multiple defects.

**Table 1.** Demographic and surgical data.

Patients	110 (64 M, 46 F)
Age (years)	59.8 ± 16.5
Follow-up (months)	13.2 ± 8.1
Application (two patients had multiple defects)	
AWR	82 (74.5%)
parastomal repair	6 (5.5%)
Inguinal hernia	18 (16.4%)
other	9 (8.2%)
VHWG grading	
Clean	67 (60.9%)
Co-morbid	15 (13.6%)
Contaminated	28 (25.5%)
Closure	
Primary	95 (86.4%)
TAR*	9 (8.2%)
Flap	6 (5.5%)

* (Transversus Abdominis Release).

Implantation sites were classified following VHWG reviewed grading system as clean (67 cases), co-morbid (15 cases), contaminated (28 cases). In most cases a primary closure supported by the biological matrix was considered sufficient (95 cases), in nine cases *transversus abdominis* release was necessary for effective closure, and six cases involved the use of a microsurgical flap.

Complications are reported in Table 2. Seven (6.4%) infections requiring antibiotics and aspiration of serous collection were recorded; one of them occurred in a patient receiving ADM for abdominal wall reinforcement following DIEP flap harvest for breast reconstruction, while three presented a concomitant fluid collection.

**Table 2.** Complications.

Complications	
defect recurrence/bulging	3 (2.7%)
Infection	7 (6.4%)
Necrosis	4 (3.6%)
seroma	4 (3.6%)
Total complicated patients	14 (12.7%)

Three patients (2.7%) presented a recurrence of the defect; one of them who received a flap-aided closure developed bulging, and one patient classified as having a contaminated field had a hernia recurrence even though they did not manifest signs of infection.

Four patients (3.6%) developed a necrosis; one of them undergoing two simultaneous surgeries and presenting a large defect (≥200 cm^2^) developed a minor post-surgical necrosis associated to serum collection and was treated conservatively. All seroma cases add up to four (3.6%).

The overall number of complicated cases is limited to 14 (12.7%). All in all, the outcomes are good, also in co-morbid and contaminated fields.

A case of EGIS matrix application for functional abdominal reconstruction after retroperitoneal sarcoma resection is represented in Figure 1.

## Discussion

In the Seventies the use of industrialized biological products entered standard clinical practice with the application of preserved animal tissues such as treated bovine *fascia*. They found application in AWR and in many other different surgical applications in which traumas or oncological resections left defects needing repair or reconstruction. Their efficacy and superiority respect to synthetic materials were described specially in cases of possible contamination more where patients skin tissue was not enough to cover the entire defect [12,13]. In fact, synthetic meshes are generally considered suitable only for clean uncontaminated implantation sites, not in contact with viscera since synthetic materials provide bacteria a surface for biofilm development, they cause chronic inflammatory reactions, and tend to cause adhesions and erosions of viscera with consequent obstructions or peritonitis [14,15]. This constitutes substantial reason for the choice of material in our practice described in this work, as the use of porcine acellular dermal matrices is nowadays to be considered a well-established technology, with a consolidated role in trunk surgery, among other disciplines, for the reconstruction, reinforcement, repair, and substitution of soft tissues in plastic and reconstructive surgery applications.

In this study, we collected 110 procedures with the implantation of EGIS^®^ ADM carried out between January 2021 and March 2023, operated by a plastic and reconstructive surgery unit and an oncological surgery unit.

Previously published applications of EGIS^®^ ADM comprise the use in trunk reconstruction surgeries (cases of abdominal wall and thoracic wall defects), reconstruction and augmentation of chest soft tissues (nipple reconstruction), abdominal wall repair, and other general surgery applications, also in cases of organs transplantations [12–17]. Its characteristics and the possibility to act as a soft tissues regenerative substitute render EGIS^®^ ADM suitable for various procedures and specialties where trunk soft tissues defects exist: general surgery, gynaecological surgery, proctological surgery, digestive surgery, and all the plastic, reconstructive and regenerative surgery procedures including those performed in trauma patients and oncological patients, even in combination with negative-pressure wound therapy (NPWT). In all applications the device resulted safe and effective for the purposes there stated. In our collection, postoperative complications rates resulted at acceptable levels, with none but surgical site infection below 5%. However, all fall within previously published literature, spanning 5.2–15%, leading us to consider our results well in line with existing data [18,19].

One patient received ADM abdominal reinforcement as a preventive measure against laparocele following harvest of a DIEP flap used for microsurgical breast reconstruction. In this case, as others published in literature, the patient’s abdomen showed a marked tissue laxity, so we considered it appropriate to prevent a possible occurrence with the insertion of a reinforcement. Having chosen a sublay placement, we opted for the use of a biological matrix as they are less prone to adhesions [14,15,20].

Byrnes et al. [21] describes the use of porcine acellular dermal matrix (XEN) for the reconstruction of the abdominal wall encountering very favourable outcomes (only 7.3 recurrence rate) in very difficult patients in which other approaches had failed [22]. Diaz-Siso et al. [18] has positive results with a non-crosslinked porcine acellular dermal matrix (XEN) in the reconstruction of complex trunk walls defects and a recurrence rate of 7.9% in a limited number of difficult cases (22 patients) [23].

Romain et al. [24] compares the use of crosslinked and non-crosslinked porcine acellular dermal matrices for a total of 39 cases of contaminated ventral hernia repair surgery. In either case the results are positive, but non-crosslinked ADM use resulted in a much lower reoperation rate (6.7% vs. 29.2%) [25].

George et al. [26] presented their cohort of 21 oncological patients requiring thoracic tumour resection and chest wall reconstruction. The reconstructive outcome was positive for all surviving patients, in fact one died due to oncological disease progression, and the remaining 20 patients only accused two complications (2 infections) not related to the use of the matrix [27].

Gravey et al. presents long-term studies on the use of ADM in AWR and, excluding bridged repair as a technique known for the high incidence of failure regardless of the type of device used, they record outcomes comparable to the use of synthetic meshes. With a Kaplan-Meier chart, cumulative hernia recurrence rates were calculated as 6.4% at 3-year follow-up and 8.3% at 5-year follow-up for xenogeneic ADMs. Considering the shorter follow-up we recorded, our experience appears to be in line with these data presenting a 2.7% rate of recurrences or laxities of the repaired site, envisioning a positive evolution in time [28].

In the last three decades, the major novelty regarding synthetic nets and meshes is the proliferation of devices made of resorbable plastics. In general surgery, their characteristic of gradually dissolving makes them preferable to permanent polymers devices for the use in clean sites where important tissue losses are not present, providing temporary support for surgical access healing after open abdominal surgery [29]. Biological matrices brands as well have largely increased: more than twenty brands are now applied in breast reconstruction [30]. Le Blanc in fact counted 20 biological prosthetic implants for hernioplasty in the USA already in 2018 [31]. The wide range of choice of implants probably creates confusion and no clear data exist to figure out comparatively the performance of each brand-material-shape iteration. Furthermore, some authors themselves mix biological prostheses derived from biological tissues with synthetic absorbable plastic prostheses (often unduly called ‘biosynthetic’ as if they were produced by any living organisms) drawing conclusions without due considerations [32,33]. At the same time, clinical practice is reported to benefit from the application of biological matrices in multiple publications, and our experience associates with such reports [16–19,27,28,34].

Based on the findings of our study, the use of this non-crosslinked porcine ADM is a safe and effective tool in many procedures of soft tissues repair, reinforcement, and reconstruction surgery applied to cases from different specialties contexts. Results are also promising for positive long-term outcomes, for which further studies with larger cohorts are sought.
